# Estimating the national seroprevalence of *Toxoplasma gondii* infection in pregnant women, France 2021

**DOI:** 10.1017/S0950268825100502

**Published:** 2025-09-08

**Authors:** Sara Mazzilli, Mathieu Tourdjman, Harold Noël, Anna Maisa, Isabelle Villena, Camille Le-Ray, ENP2021 study group

**Affiliations:** 1 Department of Infectious Diseases, French National Public Health Agency (Santé publique France), Saint-Maurice, France; 2 ECDC Fellowship Programme, Field Epidemiology path (EPIET), European Centre for Disease Prevention and Control (ECDC), Stockholm, Sweden; 3 National Reference Centre for Toxoplasmosis, Hospital, University Reims Champagne-Ardenne, Reims, France; 4 Centre of Research In Epidemiology and Statistics (CRESS), Obstetric, Perinatal, Paediatric Life Course Epidemiology (OPPaLE) Research Team, Université Paris Cité, Institut Santé des femmes, U1153, INSERM, INRAE, Paris, France; 5 Port-Royal Maternity Unit, Assistance Publique-Hôpitaux de Paris, Hôpital Cochin, FHU préma, Paris, France

**Keywords:** *Toxoplasma gondii*, toxoplasmosis, seroprevalence, seroconversion, pregnant woman, France

## Abstract

Toxoplasmosis during pregnancy can cause congenital malformations and fetal death. This study aimed to estimate the *Toxoplasma gondii* seroprevalence among pregnant women participating in the 2021 French national perinatal survey and identify associated factors. All women giving birth in France during the study period were invited to participate. Data collected included demographic information, nationality, socio-economic status, education level, and *Toxoplasma gondii* serological status. Women were classified as seropositive if IgG antibodies were present or if seroconversion occurred during pregnancy. Univariate and multivariate Poisson regression analyses with robust error variance were used to estimate prevalence ratios and identify factors associated with seropositivity. Among 12,612 women, the overall seroprevalence was 25.9%, and 0.22% seroconverted during pregnancy. Seroprevalence increased by 5% with every 5-year age increment and was significantly higher in the French overseas territories of Mayotte (75.0%), La Réunion (35.8%), and French Guiana (33.3%). Seroprevalence was also higher among women with lower educational levels (47.4% for primary education) and those of Sub-Saharan African nationality (52.0%). Geographic and socio-demographic variations may reflect dietary and environmental diversity. Despite declining seroprevalence in France, continued public health efforts, particularly among high-risk populations, remain critical to minimize the impact of congenital toxoplasmosis.

## Introduction

Toxoplasmosis is a parasitic infection caused by *Toxoplasma (T.) gondii*, a protozoan that infects humans and animals globally. Felids, particularly domestic cats, are the definitive hosts, while warm-blooded animals serve as intermediate hosts. The primary route of transmission to humans is through the ingestion of tissue cysts found in undercooked meat of infected animals, primarily other intermediate hosts such as pigs, sheep, and goats [1, 2]. Additionally, *T. gondii* oocysts are excreted in the faeces of infected felids and can contaminate the environment. Thus, humans can also be infected through consumption of contaminated fruits, vegetables, and water, or through contact with soil or surfaces contaminated with oocysts [3].

In the majority of cases, acquired toxoplasmosis in healthy individuals is asymptomatic or presents as a mild illness with flu-like symptoms [4]. Conversely, primary infection during pregnancy can result in vertical transmission, leading to congenital toxoplasmosis, with potentially devastating consequences for the foetus. Congenital toxoplasmosis is characterized by a spectrum of manifestations, including ocular lesions, hydrocephaly, intracranial calcifications, and hepatosplenomegaly, and can result in foetal death. Vertical transmission is estimated to occur in 20%–25% of maternal antenatal infections, particularly during the third trimester, and increases with gestational age [5]. Infected babies born to mothers contaminated during pregnancy are primarily asymptomatic, but long-term consequences of congenital toxoplasmosis can be severe, with affected infants experiencing developmental delays, visual impairments, and neurological complications even when symptoms are not immediately apparent at birth [2, 3].

France has long been considered a high-prevalence country for toxoplasmosis, with seroprevalence rates among pregnant women exceeding 80% in the 1960s [6]. The national congenital toxoplasmosis prevention programme, which includes universal screening in the first trimester, monthly screening of seronegative women, and appropriate antenatal treatment for seroconversion, has contributed to a significant reduction in maternal and congenital cases. Between 1995 and 2016, the seroprevalence among pregnant women decreased from 54% to 31%, and the incidence of seroconversion during pregnancy decreased from 5.4 to 3.1 per 1,000 pregnancies at risk (seronegative women) [7].

Although congenital toxoplasmosis has declined over the past few decades, there is still much to be understood about the factors that influence infection rates, long-term outcomes associated with the disease, and the cost-effectiveness of prevention strategies. In response to this public health challenge, France implemented a national surveillance programme for congenital toxoplasmosis (Toxosurv) in 2007 [8]. Identifying these factors is essential for refining screening protocols and prevention programmes, ensuring early detection, and ultimately reducing the burden of congenital toxoplasmosis.

This study aims to assess the current seroprevalence of toxoplasmosis among pregnant women in mainland France and French oversea territories in 2021 and to investigate the sociodemographic and regional factors associated with infection, to guide future public health initiatives.

## Methods

The National Perinatal Survey (ENP) is a periodic national cross-sectional survey. Five similar surveys were previously carried out in France in 1995, 1998, 2003, 2010, 2016, and 2021 [7, 9, 10].

### Study population and inclusion criteria

The 2021 ENP included women who gave birth in all public and private maternity hospitals and birthing centres across France. Participants were recruited on a voluntary basis over several weeks between March and December 2021 according to the schedule below:

- Mainland France and French Guiana – 1 week from 15 to 21 March 2021 or over two consecutive weeks (1 day in 2) between 8 and 28 March for maternity units with a large number of births
- La Réunion: from 15 March to 11 April 2021
- Mayotte: from 25 October to 5 December 2021
- Guadeloupe/Saint-Martin: from 15 March to 16 May 2021
- Martinique: from 15 March to 20 June 2021

The survey covers all births >22 gestational weeks of birthweight over 500 g, where the child was born alive.

### Data collection and definitions

Healthcare staff conducted a 15-min structured interview with participating women in the maternity unit, collecting data on demographics (e.g., nationality, education, and income), living conditions, antenatal care, and preventive behaviours. The interview was supplemented by information gathered from the medical records, including serological data on toxoplasmosis, pregnancy complications, the delivery process, and the health status of the mother and new-born. Women who refused to participate were asked to respond to a minimal questionnaire with a core dataset [11].

A woman was classified as seropositive if the presence of IgG antibodies was documented at the initial screening, or if seroconversion was observed during pregnancy. She was classified as seronegative if no antibodies were detected at the start of pregnancy and during subsequent prenatal screenings.

### Statistical analysis

To account for the varying survey durations across different territories, weighting adjustments for the calculation of seroprevalences were applied during the statistical analysis for comparison of these prevalences among geographical areas. The weighting factors for all mothers and new-borns in each territory were determined by the inverse of the number of survey weeks. The assigned weights were one for mothers/new-borns in Mainland France and French Guiana, and, respectively, 1/9, 1/14, 1/4, and 1/6 for those in Guadeloupe/Saint-Martin, Martinique, La Réunion, and Mayotte Islands.

Descriptive statistical analysis was performed to analyze the sample characteristics. Differences in categorical variables according to serological status were assessed using chi-squared tests with Rao and Scott’s second-order correction to account for the complex survey design. These analyses were conducted on complete cases for the variables included in each test. Due to the high proportion of missing values and the non-random pattern observed for salary, analyses involving this variable may be affected by selection bias. We retained salary in these analyses, given its theoretical relevance.

Univariate and multivariate analyses were conducted to estimate prevalence ratios (PRs) and identify factors associated with seropositivity using a Poisson regression model with robust error variance [12]. We performed a complete-case analysis, including only participants with non-missing data for all covariates in the model. The multivariate model was constructed using a backward stepwise elimination approach, retaining variables with a *p*-value <0.01 in univariate analyses. A *p*-value of 0.05 was applied for testing statistical significance.

The methodology for assessing interactions between explanatory variables in predicting the outcome involved first evaluating the main effects and interaction terms by examining the *p*-values associated with the respective regression coefficients. The significance of the interaction term was determined through likelihood ratio tests conducted between the full model (which included the interaction term) and a reduced model (which excluded the interaction term). The likelihood ratio test statistic, along with the corresponding *p*-value, was utilized to determine whether the inclusion of the interaction term significantly improved the model fit. To estimate the interaction effect between the explanatory variables, marginal means were computed for each combination of the variables using model-derived predictions. The relative effect was expressed as a PR with confidence intervals (CIs), calculated as the ratio of the predicted outcome for each category to the predicted outcome in the reference category.

All statistical analyses were performed using R version 4.3.1.

### Ethical approval

The ENP is a public statistical survey that received several official approvals: the National Council on Statistical Information (Conseil National de l’Information Statistique) endorsed it on 14 October 2019, approval no. 141/H030; the Committee of Ethics and Scientific Research, Studies and Evaluations (Comité d’Ethique et Scientifiques pour les Recherches, les Études et les Évaluations on 12 June 2020); the Committee for the Protection of Persons (Comité de Protection des Personnes) issued a favorable opinion on 7 July 2020; and the French Data Protection Authority (Commission Nationale de l’Informatique et des Liberté) authorized it under DR-2020-391 on 31 December 2020. It received a label of general interest and statistical quality from the Label Committee (the Comité du Label) (Visa No. 2021X701SA, decree of 23 November 2020). All data used for analysis were anonymized to ensure participant confidentiality.

## Results

### Descriptive results

**Figure 1.**
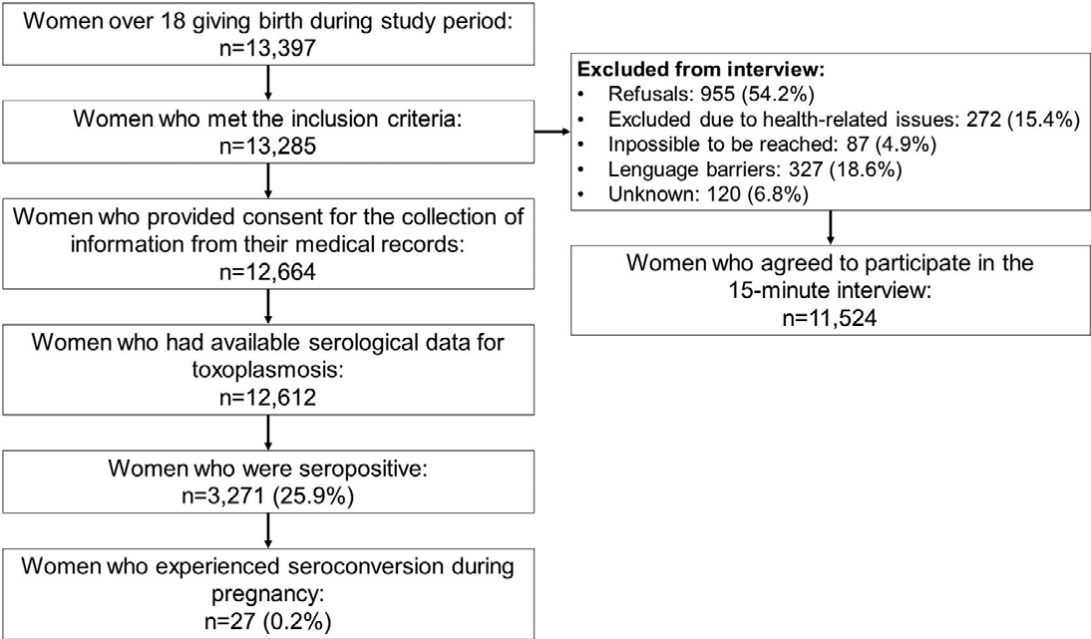
Flowchart of participant selection and seroprevalence of toxoplasmosis among pregnant women in France, 2021.

With weighting adjustments applied, a total of 13,397 women over the age of 18 gave birth during the study period, of whom 13,285 met the inclusion criteria. A total of 11,524 women agreed to participate in the structured 15-min interview. Among the 1,761 women who did not participate, 955 (54.2%) declined to take part. An additional 272 (15.4%) were excluded due to health-related issues affecting either the mother or the infant at the time of the interview. Another 87 (4.9%) could not be reached when contacted, and 327 (18.6%) were not interviewed owing to language barriers. Finally, for 120 women (6.8%) the reason for exclusion remains unknown (Figure 1).

**Table 1.** Demographic and socioeconomic characteristics of participants of the ENP, with weighting adjustments applied, France 2021

Characteristic	Participants N = 13,285	
	n	%
Seropositivity status		
Seronegative	9,341	74.1
Seropositive	3,271	25.9
Unknown	672	-
Age		
18–24	1,529	12.0
25–29	4,519	35.5
30–34	3,559	28.0
35–39	2,429	19.1
≥40	692	5.4
Unknown	557	-
Nationality		
French	9,608	83.4
Other Europe	421	3.7
North Africa	595	5.2
Sub-Saharan Africa	610	5.3
Other	281	2.4
Unknown	1,770	-
Education		
Primary or less	240	2.1
Middle school	2,036	17.7
High school diploma	2,538	22.1
University or equivalent	6,691	58.2
Unknown	1,779	-
Net monthly household salary (EUR)		
< 1,500	813	15.3
1,500–4,000	3,416	64.3
> 4,000	1,081	20.4
Unknown	7,975	-
Geographical area		
Île-de-France	3,094	23.3
Auvergne-Rhône-Alpes	1,574	11.8
Bourgogne-Franche-Comté	482	3.6
Bretagne	561	4.2
Centre-Val de Loire	458	3.4
Corse	48	0.4
Grand Est	920	6.9
Hauts-de-France	1,121	8.4
Normandie	572	4.3
Nouvelle-Aquitaine	1,006	7.6
Occitanie	1,109	8.3
Provence-Alpes-Côte d’Azur	911	6.9
Pays de la Loire	742	5.6
Guadeloupe/Saint-Martin	84	0.6
Martinique	57	0.4
French Guiana	119	0.9
La Réunion	270	2.0
Mayotte	156	1.2

A total of 12,664 women provided consent for the collection of information from their medical records. Among these, serological data for toxoplasmosis were available for 12,612 women, representing 94.9% of the 13,285 eligible participants. The median age of the participants was 31 years (IQR: 27–34). The majority were of French nationality (9,608, 83.4%), followed by African nationalities (1,205, 10.5%) and European nationalities excluding French (421, 3.7%). Most respondents (58.2%) had a university-level or higher education, and 64.3% reported a monthly household income between 1,500 and 4,000 euros. The majority of respondents (12,598, 94.8%) resided in mainland France (Table 1).

**Table 2.** Demographic and socioeconomic characteristics of participants of the ENP by seropositivity status and ENP by seroconversion during pregnancy, with weighting adjustments applied, France 2021

Characteristic	Seronegative N = 9,341		Seropositive N = 3,271		p-value^a^	Seronegative or seroconversion before pregnancy N = 12,585		Seroconversion during pregnancy N = 27		p-value^a^
	n	%	n	%		n	%	n	%	
Age					<0.001					0.4
18–24	1,211	80.0	303	20.0		1,509	99.7	5	0.3	
25–29	3,518	78.6	960	21.4		4,471	99.8	7	0.2	
30–34	2,637	74.7	891	25.3		3,522	99.8	6	0.2	
35–39	1,587	66.0	818	34.0		2,397	99.7	8	0.3	
≥40	383	56.1	299	43.9		681	99.8	1	0.2	
Unknown	5	-	-	-		5	-	0	-	
Nationality					<0.001					0.8
French	7,237	76.0	2,285	24.0		9,5	99.8	23	0.2	
Other Europe	321	77.1	96	22.9		416	100.0	0	0.0	
North Africa	377	64.3	209	35.7		585	99.8	1	0.2	
Sub-Saharan Africa	289	48.0	313	52.0		600	99.8	1	0.2	
Other	205	74.2	72	25.8		276	99.6	1	0.4	
Unknown	912	-	296			1,208	-	1	-	
Education					<0.001					0.3
Primary or less	124	52.6	112	47.4		235	99.8	1	0.2	
Middle school	1,482	73.3	540	26.7		2,015	99.6	7	0.4	
High school diploma	1,885	75.0	627	25.0		2,505	99.7	7	0.3	
University or equivalent	4,934	74.5	1,692	25.5		6,614	99.8	11	0.2	
Unknown	916	-	300	-		1,216	-	1	-	
Net monthly household salary (EUR)					<0.001					0.9
< 1,500	538	67.0	264	33.0		800	99.7	2	0.3	
1,500–4,000	2,621	77.3	770	22.7		3,383	99.8	8	0.2	
> 4,000	762	71.0	312	29.0		1,071	99.7	3	0.3	
Unknown	5,421	-	1,925	-		7,331	-	14	-	
Geographical area					<0.001					
Île-de-France	2,074	70.9	853	29.1		2,924	99.9	3	0.1	
Auvergne-Rhône-Alpes	1,201	79.8	304	20.2		1,501	99.7	4	0.3	
Bourgogne-Franche-Comté	359	76.7	109	23.3		468	100.0	0	0.0	
Bretagne	419	77.2	124	22.8		542	99.8	1	0.2	
Centre-Val de Loire	325	74.5	111	25.5		436	100.0	0	0.0	
Corse	40	83.3	8	16.7		48	100.0	0	0.0	
Grand Est	715	82.5	152	17.5		866	99.9	1	0.1	
Hauts-de-France	811	76.8	245	23.2		1,052	99.6	4	0.4	
Normandie	421	75.7	135	24.3		556	100.0	0	0.0	
Nouvelle-Aquitaine	673	70.9	276	29.1		947	99.8	2	0.2	
Occitanie	745	71.9	291	28.1		1,035	99.9	1	0.1	
Provence-Alpes-Côte d’Azur	653	74.6	222	25.4		871	99.5	4	0.5	
Pays de la Loire	537	77.6	155	22.4		690	99.7	2	0.3	
Guadeloupe/Saint-Martin	50	66.6	25	33.4		75	99.9	0	0.1	
Martinique	37	69.0	16	31.0		53	99.7	0	0.3	
French Guiana	78	66.7	39	33.3		115	98.3	2	1.7	
La Réunion	165	64.2	92	35.8		256	99.7	1	0.3	
Mayotte	38	25.0	114	75.0		150	98.6	2	1.4	

a Pearson’s X^2^: Rao & Scott adjustment.

Among study participants with available serological data, 3,271 (25.9%) were seropositive, of whom 27 (0.2%) experienced seroconversion during pregnancy (Table 2).

**Table 3.** Prevalence ratios of seropositivity by sociodemographic and geographic characteristics: results from the univariate and multivariate analysis (coefficients of the multivariate model are calculated taking the interaction Education*Nationality into account), France, ENP 2021

Characteristic	Univariate			Multivariate		
	Crude prevalence ratio	Confidence Interval 95%	p-value	Adjusted prevalence ratio	Confidence Interval 95%	p-value
Age						
18–24	0.96	0.94–0.98	<0.001	0.93	0.90–0.96	<0.001
25–29	0.97	0.96–0.99	<0.001	0.96	0.94–0.98	<0.001
30–34	Ref.	Ref.	Ref.	Ref.	Ref.	Ref.
35–39	1.07	1.05–1.09	<0.001	1.05	1.02–1.08	<0.001
≥40	1.15	1.12–1.18	<0.001	1.13	1.08–1.18	<0.001
Nationality						
French	Ref.	Ref.	Ref.	Ref.	Ref.	Ref.
Other Europe	0.99	0.96–1.03	0.62	0.97	0.91–1.04	0.44
North Africa	1.09	1.06–1.13	<0.001	1.07	1.00–1.14	0.05
Sub-Saharan Africa	1.23	1.20–1.26	<0.001	1.10	1.01–1.19	0.03
Other	1.01	0.98–1.06	0.47	0.97	0.90–1.06	0.52
Education						
Primary or less	1.17	1.13–1.22	<0.001	1.22	1.04–1.42	0.01
Middle school	1.01	0.99–1.03	0.27	1.01	0.98–1.04	0.42
High school diploma	1.00	0.98–1.01	0.56	1.00	0.98–1.03	0.76
University or equivalent	Ref.	Ref.	Ref.	Ref.	Ref.	Ref.
Net monthly household salary (EUR)						
< 1,500	Ref.	Ref.	Ref.	Ref.	Ref.	Ref.
1,500–4,000	0.92	0.90–0.95	<0.001	0.97	0.94–1.00	0.03
> 4,000	0.97	0.94–1.00	0.06	1.00	0.96–1.04	0.98
Geographical area						
Île-de-France	Ref.	Ref.	Ref.	Ref.	Ref.	Ref.
Auvergne-Rhône-Alpes	0.93	0.91–0.95	<0.001	0.95	0.93–0.99	<0.001
Bourgogne-Franche-Comté	0.95	0.92–0.99	0.01	0.96	0.91–1.01	0.11
Bretagne	0.95	0.92–0.98	<0.001	0.97	0.93–1.02	0.27
Centre-Val de Loire	0.97	0.94–1.01	0.11	0.99	0.94–1.04	0.74
Corse	0.90	0.82–0.99	0.03	0.93	0.81–1.06	0.27
Grand Est	0.91	0.89–0.93	<0.001	0.91	0.88–0.94	<0.001
Hauts-de-France	0.95	0.93–0.98	<0.001	1.00	0.96–1.05	0.93
Normandie	0.96	0.93–0.99	0.02	1.00	0.96–1.05	0.93
Nouvelle-Aquitaine	1.00	0.97–1.03	0.98	1.05	1.01–1.09	0.03
Occitanie	0.99	0.97–1.02	0.52	0.99	0.95–1.03	0.59
Provence-Alpes-Côte d’Azur	0.97	0.95–1.00	<0.001	0.97	0.93–1.01	0.15
Pays de la Loire	0.95	0.92–0.98	<0.001	0.94	0.91–0.98	0.01
Guadeloupe/Saint-Martin	1.03	1.00–1.06	0.03	1.06	1.00–1.12	0.04
Martinique	1.01	0.99–1.04	0.33	1.01	0.97–1.06	0.54
French Guiana	1.03	0.97–1.10	0.34	1.02	0.92–1.14	0.70
La Réunion	1.05	1.03–1.08	<0.001	1.07	1.03–1.12	<0.001
Mayotte	1.36	1.33–1.38	<0.001	1.27	1.21–1.33	<0.001

No significant differences were observed between women who seroconverted during pregnancy and those who were seronegative or had seroconverted before pregnancy, across age, nationality, education level, household income, or geographic region (all *p* >0.05, Rao–Scott adjusted Pearson χ^2^ test).

Seroprevalence of toxoplasmosis increased progressively with age, ranging from 20.0% among participants aged 18–24 years to 43.9% among those aged 40 years and older.

Among women with French nationality, 24.0% were seropositive, while in individuals from other European countries, the seroprevalence was 22.9%. Among participants from North Africa, the seroprevalence was 35.7%, and among participants from Sub-Saharan African countries, 52.0% were seropositive. Among participants with primary-level education or less, toxoplasmosis seroprevalence was 47.4%. This seroprevalence was lower among individuals with middle school education (26.7%) and higher secondary education (25.0%). The proportion of seropositive individuals among those with a monthly household income of less than €1,500 was 33.0%.

Among participants with an income between €1,500 and €4,000, seroprevalence was 22.7% and among those earning more than €4,000, it was 29.0%.

**Figure 2.**
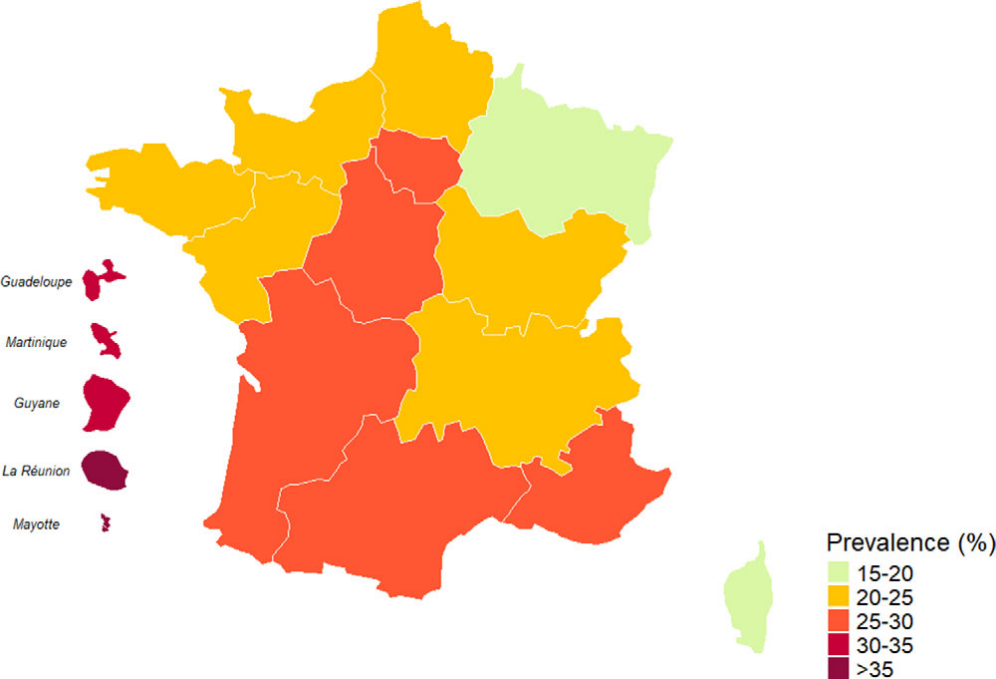
Geographical differences in seroprevalence (%) of toxoplasmosis, ENP, France 2021.

In mainland France, the highest proportion of seropositive individuals was found in Île-de-France (29.1%) and Nouvelle-Aquitaine 29.1% regions, while the lowest proportions were recorded in Grand Est (17.5%) and Corsica (16.7%) regions. Seropositivity was particularly high, in the French overseas territories, with Mayotte showing 75% seroprevalence, followed by La Réunion (35.8%) and French Guiana (33.3%) (Table 2 and Figure 2).

**Table 4.** Distribution of participants’ education level among the ENP study population, France, 2021

	Education								
	Primary or lessN = 240^a^		Middle schoolN = 2,036^a^		High school diplomaN = 2,538^a^		University or equivalentN = 6,691^a^		p-value^b^
	n	%	n	%	n	%	n	%	
Nationality									<0.001
French	38	(0.4)	1,648	(17.2)	2,063	(21.5)	5,852	(61.0)	
Other Europe	21	(5.1)	72	(17.5)	109	(26.2)	212	(51.2)	
North Africa	17	(2.9)	111	(18.7)	159	(26.8)	307	(51.7)	
Sub-Saharan Africa	150	(24.7)	151	(24.7)	141	(23.1)	168	(27.5)	
Other	14	(4.9)	55	(19.5)	63	(22.6)	148	(52.9)	
Unknown	0	-	0	-	3	-	4	-	

a n (%).

b chi-squared test with Rao & Scott’s second-order correction.

**Table 5.** Adjusted prevalence ratios (APR) of seropositivity by nationality and education level, showing the interaction effect

	Education							
	Primary or less		Middle school		High school diploma		University or equivalent	
	APR	CI 95%	APR	CI 95%	APR	CI 95%	APR	CI 95%
Nationality								
French	1.13	1.01–1.25	0.98	0.96–0.99	0.98	0.96–0.99	Ref.	Ref.
Other Europe	1.04	0.89–1.21	1.05	0.97–1.14	0.96	0.90–1.02	0.96	0.92–1.00
North Africa	1.08	0.91–1.28	1.10	1.03–1.18	1.08	1.02–1.14	1.08	1.04–1.13
Sub-Saharan Africa	1.24	1.18–1.29	1.27	1.21–1.32	1.26	1.20–1.33	1.11	1.05–1.17
Other	1.02	0.86–1.21	1.13	1.04–1.24	1.07	0.99–1.17	0.93	0.88–0.98

### Results from multivariate analysis

In the multivariate analysis, toxoplasmosis seroprevalence increased with age. Participants aged 18–24 years and 25–29 years had lower prevalence rates of seropositivity compared with the reference group (30–34 years), with adjusted prevalence ratios (APR) of 0.93 (95% CI: 0.90–0.96) and 0.96 (95% CI: 0.94–0.98), respectively. On the other hand, individuals aged 35–39 years and those aged 40 years or older had progressively higher seroprevalence, with APRs of 1.05 (95% CI: 1.02–1.08) and 1.13 (95% CI: 1.08–1.18), respectively (Table 3).

Seroprevalence of toxoplasmosis was higher among participants originating from North Africa compared with those of French nationality (APR = 1.07; 95% CI: 1.00–1.14), and among participants from Sub-Saharan Africa (APR = 1.10; 95% CI: 1.01–1.19; *p* = 0.03). No significant differences were observed for participants from other European countries (APR = 0.97; 95% CI: 0.91–1.04) or other regions (APR = 0.97; 95% CI: 0.90–1.06) compared with participants of French nationality. Participants with primary or lower education had a higher adjusted seroprevalence of toxoplasmosis compared with those with tertiary education (APR = 1.22; 95% CI: 1.04–1.42; *p* = 0.01). No significant differences were observed for participants with middle school education (APR = 1.01; 95% CI: 0.98–1.04; *p* = 0.42) or upper secondary education (APR = 1.00; 95% CI: 0.98–1.03; *p* = 0.76). Participants with a net monthly household salary between €1,500 and €4,000 had a slightly lower adjusted seroprevalence of toxoplasmosis compared with those earning less than €1,500 (APR = 0.97; 95% CI: 0.94–1.00; p = 0.03). Geographically, participants from Auvergne-Rhône-Alpes (APR = 0.95, 95% CI: 0.93–0.99, *p* < 0.001), Grand Est (APR = 0.91, 95% CI: 0.88–0.94, *p* < 0.001), and Pays de la Loire (APR = 0.94, 95% CI: 0.91–0.98, *p* = 0.01) had lower toxoplasmosis seroprevalence compared with those from Île-de-France, which was the reference region. Conversely, participants from Mayotte had a significantly higher seroprevalence (APR = 1.27, 95% CI: 1.21–1.33, *p* < 0.001), as did participants from La Réunion (APR = 1.07, 95% CI: 1.03–1.12, *p* < 0.001)).

The likelihood ratio test revealed a significant interaction between education and nationality (*p* < 0.001), which suggests a varying relationship between education and toxoplasmosis seroprevalence depending on nationality; for this reason, the multivariate model presented in Table 3 includes the interaction term Education*Nationality – data not shown. The interactions among the other variables in the analysis did not reach statistical significance and were therefore not included in the final model to maintain parsimony and focus on the most relevant factors influencing seroprevalence.

French participants predominantly have a university education (61%), followed by those with higher secondary education (21.5%). Other European and North African participants show a similar trend, with most having completed higher secondary education (around 26%) and a high proportion attaining university education (51%–52%). Sub-Saharan African participants have a more balanced distribution across education levels, with significant proportions completing middle school or primary education (24.7%) (Table 4).

For French individuals, those with primary or lower education had a significantly higher likelihood of seropositivity (APR = 1.13, 95% CI: 1.01–1.25) compared with the university education reference group (French participants with tertiary education). For North and sub-Saharan participants, the likelihood of being seropositive for toxoplasmosis was consistently higher compared with the reference group across all education levels (Table 5).

## Discussion

In this study, the estimated seroprevalence of *T. gondii* infection among pregnant women in France in 2021 was 25.9%. The findings indicate a marked decrease in seroprevalence compared with historical data, with a previous ENP estimate of 31.3% in 2016 [7]. This declining trend aligns with global patterns observed in developed countries, likely reflecting improved hygiene, dietary habits, and reduced exposure to risk factors [2].

As in previous ENPs, we identified significant geographic and sociodemographic disparities in toxoplasmosis seroprevalence. Higher rates observed in French overseas territories, particularly in Mayotte (75.0%) and La Réunion (35.8%), may be attributed to differing environmental conditions, dietary practices, and socioeconomic factors [13]. Similar findings have been reported in studies from tropical and subtropical regions, where warm and humid climates facilitate the survival of *T. gondii* oocysts in the environment [4]. Notably, compared with the 2016 ENP [7], a substantial decline in seroprevalence was observed across all overseas territories except Mayotte, where rates have remained persistently high. Specifically, seroprevalence decreased from 37.3% to 33.4% in Guadeloupe, from 41.7% to 33.3% in French Guiana, from 44.9% to 35.8% in La Réunion, and from 43.7% to 31.0% in Martinique, while in Mayotte the prevalence remained virtually unchanged (76.0% in 2016 vs. 75.0% in 2021).

Age is significantly associated with toxoplasmosis, with seroprevalence increasing progressively from 20.0% in the 18–24 age group to 43.9% among women aged ≥40 years. This pattern is consistent with the cumulative nature of exposure to *T. gondii* over time [3]. The association between lower education levels and higher seroprevalence (47.4% for primary education or less) suggests the role of health literacy and access to preventive measures, and is consistent with previous studies [1, 7, 14, 15].

The study also underscores the importance of considering nationality and socioeconomic status in the risk for *T. gondii* infection. Women of African nationalities and those with lower household incomes had a higher rate of seropositivity, suggesting that public health strategies could be tailored to these at-risk groups [5]. Furthermore, the significant interaction between education and nationality emphasizes the complex interplay of cultural and socioeconomic determinants in infection risk. Importantly, women of Sub-Saharan African nationalities consistently demonstrated the highest seroprevalence rates across all education levels, suggesting that factors associated with nationality, such as past environmental exposures, cultural practices, or structural inequalities, may exert a stronger influence on infection risk than educational attainment alone.

Our findings from France align with a growing body of international literature highlighting the role of socioeconomic status and nationality in shaping the risk of *T. gondii* infection. Similar to the elevated seroprevalence observed among women of African nationality and those with lower household income in our study, data from the United States have shown disproportionately higher infection rates among foreign-born individuals and ethnic minorities, particularly in economically disadvantaged groups [16]. In Brazil, women with lower educational attainment and limited access to healthcare exhibit higher seropositivity [17], while in Iran, rural residents and those with lower levels of formal education are consistently more affected [18].

In our study, the incidence of seroconversion during pregnancy was 0.22%, which is lower than the 3.1 per 1,000 pregnancies at risk (0.31%) reported by Robinson et al. in 2016, indicating a continued decline in new infections among pregnant women [7].

The decreasing seroprevalence and seroconversion of *T. gondii* among pregnant women in France raise questions about the current universal screening programme cost-effectiveness. Several studies have evaluated the economic viability of prenatal screening for toxoplasmosis. A Finnish study conducted in 1992 demonstrated that systematic serological screening during pregnancy reduced total annual costs of care by 25% compared with no screening, suggesting that such programmes are economically worthwhile even in countries with low incidence [19]. Similarly, a 1996 Canadian analysis concluded that a carefully planned screening programme for detecting and treating infants infected with *T. gondii* during pregnancy is cost-effective, as the cost of delivering the programme was less than half of the cost of providing long-term care for children born with congenital toxoplasmosis [20]. Additionally, Prusa et al. demonstrated that the Austrian national prenatal screening programme generated substantial cost savings, estimating total lifetime costs of €103 per birth under screening compared to €426 per birth without screening [21]. More recently, a 2019 study compared prenatal and neonatal screenings using a decision-analytic model based on French guidelines. The study found that prenatal screening is cost-effective compared to neonatal screening in moderate prevalence areas, with an incremental cost-effectiveness ratio indicating that €14,826 would be required to avoid one adverse event (liveborn with congenital toxoplasmosis, foetal loss, neonatal death, or pregnancy termination) [22].

This study has several limitations that should be acknowledged. The cross-sectional design captures seroprevalence at a single point in time, limiting the ability to assess temporal changes or causality. However, the study methods and design remained largely consistent across the different ENPs, supporting the comparability of findings over time. Sociodemographic information was self-reported, which may introduce reporting biases or inaccuracies. However, data were collected using a standardized questionnaire administered by trained interviewers, which likely helped minimize misclassification and improve the reliability of the information gathered.

Related to missing data, a substantial proportion of missing values was observed in the salary variable, and the pattern of missingness was not completely at random. Specifically, missing salary information was more frequent among individuals with lower educational attainment and those who were seropositive for *T. gondii.* This suggests the possibility of reporting bias, likely due to perceived social stigma or privacy concerns around income disclosure. We conducted a complete-case analysis in both bivariate and multivariable models. While this approach is commonly used, it may introduce selection bias and reduce statistical power. In particular, the association between low household income and *T. gondii* seropositivity might be underestimated due to the non-random exclusion of individuals with missing data. Although we retained salary in the models given its theoretical and empirical relevance, results should be interpreted with caution.

Despite adjusting for known confounders, there may be unmeasured variables influencing the observed associations, such as dietary habits or contact with animals. Furthermore, the findings of this national cross-sectional study may not be generalizable to other populations or regions with different environmental exposures, healthcare systems, or cultural practices.

In conclusion, our study shows a reduction in the seroprevalence of toxoplasmosis among pregnant women in France from 2016 to 2021. This declining trend likely reflects improved hygiene, dietary habits, and reduced exposure to risk factors. Evidence from previous studies indicates that prenatal screening strategies are not only cost-effective but also effective in reducing the incidence of congenital toxoplasmosis through early detection and treatment. However, continued public health efforts, focusing on education and prevention, particularly among vulnerable populations, remain critical to further minimizing the impact of congenital toxoplasmosis.
